# Genome-wide association analysis and accuracy of genome-enabled breeding value predictions for resistance to infectious hematopoietic necrosis virus in a commercial rainbow trout breeding population

**DOI:** 10.1186/s12711-019-0489-z

**Published:** 2019-08-28

**Authors:** Roger L. Vallejo, Hao Cheng, Breno O. Fragomeni, Kristy L. Shewbridge, Guangtu Gao, John R. MacMillan, Richard Towner, Yniv Palti

**Affiliations:** 1 National Center for Cool and Cold Water Aquaculture, Agricultural Research Service, United States Department of Agriculture, Kearneysville, WV USA; 2 Department of Animal Science, University of California, Davis, CA USA; 3 Department of Animal Science, University of Connecticut, Storrs, CT USA; 4 Research Division, Clear Springs Foods Inc., Buhl, ID USA; 5 Gen Tec Consulting, Fayette, ID USA

## Abstract

**Background:**

Infectious hematopoietic necrosis (IHN) is a disease of salmonid fish that is caused by the IHN virus (IHNV). Under intensive aquaculture conditions, IHNV can cause significant mortality and economic losses. Currently, there is no proven and cost-effective method for IHNV control. Clear Springs Foods, Inc. has been applying selective breeding to improve genetic resistance to IHNV in their rainbow trout breeding program. The goals of this study were to elucidate the genetic architecture of IHNV resistance in this commercial population by performing genome-wide association studies (GWAS) with multiple regression single-step methods and to assess if genomic selection can improve the accuracy of genetic merit predictions over conventional pedigree-based best linear unbiased prediction (PBLUP) using cross-validation analysis.

**Results:**

Ten moderate-effect quantitative trait loci (QTL) associated with resistance to IHNV that jointly explained up to 42% of the additive genetic variance were detected in our GWAS. Only three of the 10 QTL were detected by both single-step Bayesian multiple regression (ssBMR) and weighted single-step GBLUP (wssGBLUP) methods. The accuracy of breeding value predictions with wssGBLUP (0.33–0.39) was substantially better than with PBLUP (0.13–0.24).

**Conclusions:**

Our comprehensive genome-wide scan for QTL revealed that genetic resistance to IHNV is controlled by the oligogenic inheritance of up to 10 moderate-effect QTL and many small-effect loci in this commercial rainbow trout breeding population. Taken together, our results suggest that whole genome-enabled selection models will be more effective than the conventional pedigree-based method for breeding value estimation or the marker-assisted selection approach for improving the genetic resistance of rainbow trout to IHNV in this population.

## Background

Infectious hematopoietic necrosis (IHN) is an economically important disease of salmonid fish that is caused by IHN virus (IHNV), which is a single-stranded negative-sense RNA rhabdovirus [1]. IHNV is endemic to the Pacific Northwest in North America [2] and has spread throughout continental Europe, China, and Japan [3–5]. IHNV is infectious to Pacific salmon and trout *(Oncorhynchus* spp.), as well as to Atlantic salmon (*Salmo salar*) [2]. Under intensive conditions of aquaculture, IHNV can cause significant mortality and losses at nearly all stages of production [6, 7]. Currently, there is no proven and cost-effective method for IHNV prevention or treatment. Thus, the development of rainbow trout strains with genetic resistance to IHNV can aid in improving animal welfare and in decreasing the economic losses that are caused by this highly infectious disease to aquaculture production.

The additive genetic basis for IHNV resistance is evident from the moderate estimates of heritability for IHNV survival status *(*$h^{2}$ = 0.23 − 0.55) and survival days *(*$h^{2}$ = 0.02 − 0.20) in a steelhead trout (*O. mykiss*) population [2]. These results suggest that resistant rainbow trout strains can be developed using family-based selective breeding methods. However, to date, selecting and developing strains with improved resistance to IHNV in rainbow trout has not been successful. Phylogenetic and nucleotide sequence analyses of 84 IHNV isolates have revealed an unusually high genetic diversity of IHNV in trout aquaculture, making the process of selecting IHNV-resistant strains difficult [8]. Nonetheless, selective breeding of a rainbow trout strain for resistance to IHNV has been implemented at the Clear Springs Foods Inc. breeding program since the year 2000 [9]. Between 2000 and 2016, the selection differential for resistance to IHNV has been on average 10% for the last eight generations and the mortality rate in challenge trials has decreased on average by 3% per generation (Richard Towner, unpublished results).

Few quantitative trait loci (QTL) mapping studies have been conducted to identify genetic polymorphisms that are associated with IHNV resistance in rainbow trout. A number of moderate-large-effect QTL associated with IHNV resistance were found on 12 rainbow trout chromosomes using linkage analysis [3, 10–12] and genome-wide association studies (GWAS) [9] methods. However, these previous QTL mapping studies had several limitations. First, they performed only single-marker association (SMA) tests using single-regression or linear mixed models. A key concern with SMA compared to the multiple-marker association test model is that it ignores both the information that is contained in the joint distribution of all markers [13, 14] and the linkage disequilibrium (LD) between neighboring loci [15, 16]. Consequently, with the multi-marker model, a weak signal may be more apparent when other QTL are accounted for, and a false signal may be weakened by the inclusion of a stronger signal from a real QTL in the model [17, 18]. Second, most of the reported QTL for IHNV resistance were identified using linkage-based methods and GWAS was performed within individual segregating families, using relatively small samples and consequently with low QTL detection power. Third, the previous studies used low-density genotyping platforms and did not have a reference genome sequence for accurate prediction of the order and physical proximity of the tested markers.

There is ambiguity on the best computational algorithm when using multiple-regression based models in GWAS and genomic selection (GS) studies because the genetic architecture of the trait and the population structure can have a major impact on power to detect marker effects and on the accuracy of genomic predictions. Therefore, it is important to compare the results from the best available computational methods when elucidating the genetic architecture of a complex disease trait for the first time in a population. This approach will ensure the effective discovery of QTL underlying the genetic basis of the disease under study, and better control of the type I error rate, which is often high in GWAS.

In multiple-regression based GWAS models that fit all single nucleotides polymorphisms (SNPs) with high quality genotypes, the genomic best linear unbiased prediction (GBLUP) method assumes that all SNPs have a non-zero contribution to the variance of the trait of interest, with equal variance for each SNP, and that the distribution of the SNP effects follows a normal distribution [19–21]. In addition, the single-step GBLUP (ssGBLUP) method was developed, which combines the pedigree-based (${\mathbf{A}}$) and genomic relationship (${\mathbf{G}}$) matrices into the ${\mathbf{H}}$ relationship matrix [22, 23]. Conversely, the Bayesian variable selection model assumes that the genetic variance of the trait is explained by a relatively small number of loci, each with a small-moderate or large effect [15, 21, 24–26]. Based on the underlying assumptions of these models, the GBLUP model is expected not to perform as well as the Bayesian variable selection model when the genetic architecture of the trait is not predominantly polygenic. For that reason, the GBLUP method was extended to the weighted ssGBLUP (wssGBLUP) method, which mimics the Bayesian variable selection model by fitting all SNPs in the multiple-regression model but assigning differential weights to the SNPs based on the individual variance of each SNP effect [27]. More recently, Bayesian variable selection models that use single-step methods have been developed [18, 28–30], including the single-step Bayesian multiple regression (ssBMR) method [18, 29].

The development of these GWAS methods, along with the development of the rainbow trout 57 K SNP array [31], a dense genetic linkage map with ~ 48 K SNPs [32], and the release of the chromosome-level rainbow trout reference genome (GenBank Assembly Accession GCA_002163495.1) [33] have recently provided the essential tools to identify genomic regions that are associated with IHNV resistance and perform genome-enabled selection for resistance against IHNV in rainbow trout. The main objectives of this study, therefore, were to (1) identify QTL or genomic regions that are associated with IHNV resistance; (2) determine the genetic architecture of resistance to IHNV; (3) compare the QTL detected by the wssGBLUP and ssBMR methods; and (4) evaluate the accuracy of genomic predictions for resistance to IHNV using cross-validation analysis. All analyses were conducted using data from a commercial rainbow trout breeding population.

## Methods

### Ethics statement

This study used rainbow trout fin clips that were collected after controlled exposure to IHNV, as part of a selective breeding program at the Clear Springs Foods Inc. research facility. As farm animals used in a commercial breeding program, these fish are exempted from regulation under the US Animal Welfare Act and were therefore not subject to oversight by an Institutional Animal Care and Use Committee or other such ethics committee. This exemption is defined in US Code title 7, chapter 54, section 2132g. However, experimentation and handling were conducted in accordance with US government principles for the use and care of vertebrate animals used in testing, research, and training, which includes provisions to minimize animal suffering. Specific measures for amelioration of animal suffering during the fish pathogen testing (described in detail in the section “Fish rearing and IHNV challenge”) included minimization of handling, maintenance of optimal water temperature and oxygen saturation, and the fish were fed a standard fish meal diet to satiation daily. Fish near death with severe symptoms of infection during the observation period were removed and euthanized (by immersion in a lethal dose of MS222) before collection of fin tissue to minimize suffering. After the 3-week observation period, surviving fish were euthanized by immersion in a lethal dose of MS222 before sampling and disposal.

### Fish rearing and IHNV challenge

Samples were collected from disease-naïve parents and their disease challenged offspring fish in brood years 2014 and 2016, respectively, by staff at the Clear Springs Foods Inc. research facility in Buhl, Idaho. Briefly, healthy fish from the previous generation were artificially spawned to produce ~ 5000 fertilized eggs from 100 year-class (YC) 2016 families. Fin tissue samples from each parent fish were collected at the time of spawning. The offspring were grown to ~ 1 g (62 days post-fertilization) and 50 fish per family were selected randomly for disease challenge and were infected with IHNV by immersion into a volume of water equivalent to 10× the total body weight of the fish in g containing 10,000 plaque-forming units of IHNV per mL for 1 h (IHNV isolate 220-90). After exposure, the fish were moved to 19-L tanks by family (50 fish/family/tank), because young and small fish cannot be labeled individually, and monitored for a 21-day period, with mortality recorded daily. Fin tissue samples were collected from mortalities during the 21-day monitoring period and survivor samples were taken at the end of the challenge. Fin clips from all fish (mortalities and survivors) were individually kept in 95% ethanol until DNA was isolated using already described protocols [34].

### Rainbow trout population used in GWAS

The fish used in the GWAS included 100 pedigreed full-sib (FS) families from year-class (YC) 2016 of the commercial breeding company Clear Springs Foods, Inc. (CSF). These 100 FS families included 41 paternal half-sib (HS) families and four maternal HS families and were generated using 78 sires and 98 dams. Fifty-nine families were generated by mating each of 59 sires with a single dam. Among the families that were generated by mating a sire with multiple dams: 17 sires were mated with two dams, one sire was mated with three dams, and another sire was mated with four dams. These YC 2016 families represented a commercial nucleus breeding population that was undergoing intensive selection for growth and IHNV resistance for the past eight generations, since 2000. The fish were evaluated for IHNV survival in the laboratory challenge, with one tank per family with an initial stocking of 50 fish per tank. The 100 families were evaluated by groups of 7 to 10 families at 11 challenge dates. After the IHNV challenge, the dataset included IHNV resistance phenotype records on *n *= 4987 fish. The associated pedigree file included 6308 records.

### IHNV resistance phenotypes

The discrete IHNV resistance phenotype survival days (DAYS), i.e. the number of days post-challenge until the fish succumbed to the disease, was recorded for all mortalities, while survivors were assigned a value of 21. Each fish also had a binary survival status (STATUS) record. The resistance phenotype STATUS had two categories: 1 for fish that died during the 21 days post challenge evaluation period; and 2 for fish that survived for the duration of the challenge. The DAYS and STATUS records were analyzed separately using the univariate GWAS models described below.

### SNP genotyping platform

The fish sampled from the CSF population were genotyped using the Rainbow Trout Affymetrix 57K SNP array (Chip) following previously described procedures [31] by a commercial service provider (AKESOgen, Norcross, GA) following the Axiom genotyping procedures described by the array manufacturer (Affymetrix). We randomly sampled five survivor offspring and five early dying offspring per family (in total N = 1000) for SNP genotyping. We also genotyped all the sires from which fin clips were available (N = 53). The dams were not sampled. The quality control (QC) bioinformatics pipeline applied to the chip-SNP genotype data was previously described [35]. Briefly, the QC pipeline filtered out the SNPs that showed a significant distortion from the expected Mendelian segregation in each FS family (Bonferroni adjusted to *P *< 0.05) and removed offspring fish that did not have matching genotypes with the parents given in the pedigree (i.e. that did not pass the pedigree check). After this initial genotype data QC, 42,045 SNPs were included in the raw chip genotype dataset.

Before conducting GWAS analyses, the raw marker genotype dataset was further QC filtered using the algorithms that are implemented in the software BLUPF90 [36]. The QC retained SNPs with a genotype calling rate higher than 0.90, minor allele frequency higher than 0.05, and with departures from Hardy–Weinberg equilibrium lower than 0.15, based on the difference between expected and observed frequency of heterozygotes. Parent-progeny pairs were tested for discrepant homozygous SNPs, and SNPs with a conflict rate higher than 1% were discarded from further analysis. Next, we determined the physical map position (GenBank Assembly Accession GCA_002163495.1) [33] of each of the QC filtered SNPs and those that did not have a physical map location were removed. After this data QC, we were left with data on 35,397 genotyped SNPs and 1044 genotyped fish (992 offspring plus 52 sires) for GWAS (Table 1).

**Table 1:** Experimental variables and genetic parameter estimates for IHNV resistance in rainbow trout

IHNV resistance phenotype^a^	Method^b^	Number of families	Phenotyped fish	Genotyped^c^		Sliding 1-Mb windows	Genetic parameter^f^			
				SNPs^d^	Fish^d^		$\sigma_{g}^{2}$	$\sigma_{f/d}^{2}$	$\sigma_{e}^{2}$	$h^{2}$
DAYS	PBLUP	100	4987	n.a.^e^	n.a.	n.a.	9.44 ± 3.22	1.77 ± 1.05	17.06 ± 1.67	0.33 ± 0.10
	ssGBLUP	100	4987	35,397	1044	1414	6.35 ± 1.25	2.40 ± 0.77	18.58 ± 0.72	0.23 ± 0.04
STATUS	PBLUP	100	4987	n.a.	n.a.	n.a.	2.22 ± 17.41	0.29 ± 1.16	1.00 ± 0.04	0.28 ± 0.14
	ssGBLUP	100	4987	35,397	1044	1414	0.81 ± 0.23	0.19 ± 0.07	1.00 ± 0.03	0.25 ± 0.07

Offspring from year-class 2016 families from the nucleus breeding population of Clear Springs Foods, Inc.

^a^IHNV resistance phenotypes: survival days after disease challenge (DAYS) and binary survival status (STATUS)

^b^Variance components analysis was performed using pedigree-based BLUP (PBLUP) and PBLUP with genomics data (ssGBLUP)

^c^Sampled fish were genotyped with the 57 K SNP array (Chip)

^d^Effective number of genotyped SNPs and fish after data quality control, respectively; the initial raw dataset had 1044 fish (sires = 52; offspring = 992) genotyped with 42,045 SNPs

^e^n.a. indicates that data are not available; the PBLUP model uses only pedigree and phenotype records in the analysis

^f^Genetic parameter estimate (± standard error): $\sigma_{g }^{2}$ is the additive genetic variance; $\sigma_{f/d}^{2}$ is the variance due to nested effects of families within challenge date; $\sigma_{e}^{2}$ is the residual error; and $h^{2}$ is the estimated narrow-sense heritability. For the binary STATUS, the heritability estimated on the underlying scale of liability was transformed to the observed scale of disease survival

### Estimation of genetic parameters for IHNV resistance

The phenotypes of DAYS and STATUS (*n* = 4987) were fit to an animal linear and a threshold model, respectively, to estimate genetic variance parameters for IHNV resistance phenotypes. The variance components analysis was performed using pedigree-based BLUP (PBLUP) and PBLUP with genomic data (ssGBLUP) under a Bayesian framework, using computer applications from the BLUPF90 family of programs [36]. The discrete data survival DAYS was analyzed using an animal linear model with the software GIBBS2F90; and the binary data survival STATUS was analyzed using an animal threshold model with the software THRGIBBS1F90. The Gibbs sampling scheme included one million iterations, of which the first 200,000 iterations were discarded; from the remaining 800,000 iterations one sample was saved from every 100 iterations, such that results from 8000 samples were used in the analysis. Proper mixing and convergence of the MCMC chains were assessed with the R package CODA [37].

Heritability for DAYS or STATUS was estimated as: $h^{2} = \sigma_{a}^{2} /\left( {\sigma_{a}^{2} + \sigma_{f/d}^{2} + \sigma_{e}^{2} } \right)$; where $h^{2}$ is the estimated narrow-sense heritability; $\sigma_{a}^{2}$ is the estimated additive genetic variance; $\sigma_{f/d}^{2}$ is the estimated variance due to the nested effect of families within the challenge date; and $\sigma_{e}^{2}$ is the estimated residual error variance. The heritability for the binary survival STATUS estimated on the underlying scale of liability using a threshold model was transformed to the observed scale of disease survival STATUS using procedures already described [35].

### GWAS with weighted single-step GBLUP

We performed GWAS with the wssGBLUP method using 1-Mb sliding SNP windows [27, 36]. In the first step, effects were calculated for individual SNPs, as shown below. Afterwards, the effects of all SNPs within a 1-Mb distance were summed and compiled for each sliding window. Briefly, the 1-Mb window slides by one SNP at a time from the first SNP until the last SNP on each chromosome and the results for SNPs that are included in the window are jointly summarized; thus, the estimates for SNP effects is a moving average of $n$ adjacent SNPs included in the 1-Mb window [36]. The choice of a 1-Mb window size was based on our recent estimate of strong LD ($r^{2}$ ≥ 0.25) spanning on average over 1 Mb in the rainbow trout genome [38]. The GWAS with wssGBLUP uses all available information on sampled fish, including pedigree, genotype, and phenotype records, including those offspring fish without genotype data, $n$ = 3995 [22, 39]. The CSF sample used in GWAS included $n$ = 4987 offspring fish from 100 YC 2016 families that had IHNV resistance records (Table 1). From these phenotyped offspring fish, a subset of 992 offspring fish and 52 sires had genotype data for 35,397 effective SNPs.

In GWAS with wssGBLUP, the weights for each SNP are 1 for the first iteration, which means that all SNPs have the same weight (i.e., single-step GBLUP). For the next iterations (2nd, 3rd, etc.), the weights are SNP-specific variances that are calculated using the estimate of the SNP allele-substitution effect from the preceding iteration and the corresponding SNP allele frequencies [27]. Estimates of SNP effects were calculated using a weighted relationship matrix, using the following equation: $\widehat{{\mathbf{u}}} = {\mathbf{DM}} '\left[ {{\mathbf{MDM}} '} \right]^{ - 1} \widehat{{\mathbf{a}}}_{{\mathbf{g}}}$, where $\widehat{{\mathbf{u}}}$ is the vector of the estimated SNP effects; ${\mathbf{D}}$ is a diagonal matrix of weights for variances of SNP effects; ${\mathbf{M}}$ is a matrix relating genotypes of each SNP to each individual; and $\widehat{{\mathbf{a}}}_{{\mathbf{g}}}$ is the estimate of the additive genetic effect for genotyped animals. The individual variance of SNP effects, which corresponds to the diagonal elements of ${\mathbf{D}}$, was estimated as [40]: $\widehat{{\varvec{\upsigma}}}_{{{\mathbf{u}},{\mathbf{i}}}}^{2} = \widehat{{\mathbf{u}}}_{{\mathbf{i}}}^{2} 2{\mathbf{p}}_{{\mathbf{i}}} \left( {1 - {\mathbf{p}}_{{\mathbf{i}}} } \right)$, where: $\widehat{\text{u}}_{\text{i}}^{ 2}$ is the square of the effect at SNP ${\mathbf{i}}$, and ${\mathbf{p}}_{{\mathbf{i}}}$ is the observed allele frequency for the second allele of SNP ${\mathbf{i}}$. In this study, we used results from the second iteration of wssGBLUP, because generally they provide the highest accuracy genomic predictions [41] and marker effects [27, 42, 43].

We fitted mixed linear and threshold models for DAYS and STATUS, respectively, using the following animal model: ${\mathbf{y}} = {\mathbf{1}}\mu + {\mathbf{Za}} + {\mathbf{Wc}} + {\mathbf{e}}$, where $1$ is a vector of 1 s, $\mu$ is the overall mean of phenotypic records, ${\mathbf{a}}$ is a vector of random individual animal effects, ${\mathbf{c}}$ is a vector of random common environment effects, ${\mathbf{e}}$ is a vector of residual effects, and ${\mathbf{Z}}$ and ${\mathbf{W}}$ are incidence matrices relating records to random animal and common environment effects in ${\mathbf{a}}$ and ${\mathbf{c}}$, respectively. The variances of ${\mathbf{a}}$, ${\mathbf{c}}$ and ${\mathbf{e}}$ are:

$$var\left[ {\begin{array}{*{20}c} {\mathbf{a}} \\ {\mathbf{c}} \\ {\mathbf{e}} \\ \end{array} } \right] = \left[ {\begin{array}{*{20}c} {{\mathbf{H}}\sigma_{a}^{2} } & 0 & 0 \\ 0 & {{\mathbf{I}}\sigma_{c}^{2} } & 0 \\ 0 & 0 & {{\mathbf{I}}\sigma_{e}^{2} } \\ \end{array} } \right],$$

where $\sigma_{a}^{2}$, $\sigma_{c}^{2}$ and $\sigma_{e}^{2}$ are additive genetic, common environment and residual variances, respectively, and ${\mathbf{H}}$ is a matrix that combines pedigree (${\mathbf{A}}$) and genomic (${\mathbf{G}}$) relationship matrices, as in Aguilar et al. [22], and its inverse as defined elsewhere [22, 39].

The full-sib fish progeny from each family were allocated to one tank for IHNV challenge evaluation, so the tank and family effects were confounded. The 100 tested families were evaluated in 11 challenge dates (date), with 7 to 10 families per date. This nested random family/date effect was used to account for the common environment effect.

The GWAS for DAYS and STATUS was also performed using Bayesian methods implemented in the BLUPF90 family of programs [36]. The GWAS for DAYS was performed with the software GIBBS2F90, and the GWAS for STATUS was performed with the software THRGIBBS1F90. The MCMC Gibbs sampling scheme and the assessment of correct mixing and convergence of the MCMC iterations were similar to those described in the section of estimation of genetic parameters.

### GWAS with single-step Bayesian multiple regression

We performed GWAS for IHNV survival DAYS with a single-step Bayesian multiple regression (ssBMR) model using 1-Mb non-overlapping SNP windows [28, 29]. The ssBMR model uses the pedigree information and all animals that had phenotype and genotype records, as in the wssGBLUP method. In ssBMR, the genotypes for non-genotyped animals are imputed explicitly given the pedigree information, and corresponding imputation residuals are fitted in the model. Thus, the GWAS analysis with ssBMR model was performed using the same phenotype and genotype data records used for wssGBLUP (Table 1). The 1-Mb window’s posterior probability of association (WPPA) with the analyzed phenotype was used to estimate the window’s proportion of false positive as $PFP = 1 - WPPA$.

In GWAS for DAYS, we fitted this Bayesian linear mixed model: ${\mathbf{y}} = {\mathbf{1}}\mu \varvec{ } + {\mathbf{Xb}} + {\mathbf{Za}} + {\mathbf{Wc}} + {\mathbf{e}}$; where ${\mathbf{X}}$ is an $n \times k$ matrix of observed or imputed genotype covariates for $k$ total number of SNPs across the genome for both genotyped and non-genotyped $n$ individuals; ${\mathbf{b}}$ is a vector of random regression coefficients of $k$ additive SNP effects; and ${\mathbf{a}}$ is a vector of random polygenic effects.

The GWAS for DAYS was performed with ssBMR using the Bayesian variable selection BayesB method (BayesB) [30] implemented in the software JWAS [44]. The BayesB method fits a mixture model to estimate marker effects, which assumes that there are two types of SNPs: a fraction of SNPs with non-zero effects $\left( {1 - \pi } \right)$ that are drawn from distributions with a marker-specific variance $\left( {\sigma_{\alpha }^{2} } \right)$, and another known fraction of SNPs $\left( \pi \right)$ that a priori have zero effect on the quantitative trait [45]. In our study, the mixture parameter $\pi$ was assumed to be known and defined to meet the condition $k \le n$; where $n$ is the number of fish with genotype records, $p$ is the effective number of SNPs, and $k = \left( {1 - \pi } \right)p$ is the number of markers sampled as having a non-zero effect that are fitted simultaneously in the Bayesian multiple regression model [16]. In this study, we tested three values for $\pi$ that met the condition $k \le n,$ 0.990, 0.995, and 0.999. Then, we used $\pi$ = 0.999 in the final GWAS because this $\pi$ value yielded a GWAS that detected the largest number of genomic windows associated with IHNV resistance and with the largest additive genetic variance.

Method BayesB uses MCMC Gibbs sampling in the GWAS analysis [16]. The Gibbs sampling scheme and diagnosis methods to assess the proper mixing and convergence of the MCMC iterations were like those used in GWAS with wssGBLUP. We did not perform GWAS using sliding SNP windows under a Bayesian framework analysis and GWAS with ssBMR for the binary STATUS because these methods have not been implemented in the software JWAS.

### Detection of QTL that are associated with IHNV resistance

The results from the analyses conducted with wssGBLUP and ssBMR were used to identify 1-Mb SNP windows that are associated with IHNV resistance. A two-step approach was used to identify QTL associated with IHNV resistance. First, the 1-Mb windows that explained additive genetic variance (EGV) higher than 2% of the total were defined as associated with IHNV resistance, and 1-Mb windows with 1% $\le$ EGV $<$ 2% were defined as windows with suggestive association. Second, to determine if neighboring or overlapping windows on the same chromosome belonged to the same QTL region, we used the following criteria: all 1-Mb windows associated with IHNV resistance that were located within a region smaller than 20 Mb and that were less than 10 Mb apart from another associated 1-Mb window were grouped into a single QTL region. We applied these fairly conservative criteria for QTL identification based on our previous experience with QTL mapping [46–48] and GWAS [49] in rainbow trout and with the aim of focusing on the QTL with the strongest signal and minimizing the type I error rate in this study.

### Accuracy and bias of breeding value predictions for IHNV resistance

The accuracy and bias of breeding value predictions for DAYS and STATUS were estimated using five-fold cross-validation (CV) analysis. Briefly, the fish with phenotype and genotype records ($n$ = 992 offspring fish) were randomly assigned to five groups (validation sets) of about 198 fish (20% of the complete dataset). The breeding value predictions for fish of the validation sets were determined one validation set at a time by using the remaining 80% of the complete dataset as the training set. In addition, the fish that had only phenotype and pedigree records ($n$ = 3995) were included in the training set. The breeding values for fish in each validation set were estimated using the PBLUP, ssGBLUP, and wssGBLUP methods. The CV analyses were performed using the BLUPF90 family of programs [36] and computer scripts that were written to automate the CV analysis (available from the authors). The DAYS and STATUS records were analyzed using the models and methods described in the section of GWAS with wssGBLUP. The Gibbs sampling scheme and diagnosis of proper mixing and convergence of the MCMC iterations were like those described in the GWAS section. We did not assess the accuracy or bias of breeding value predictions with the ssBMR method because the threshold model routine to analyze binary trait STATUS has not been implemented in the software JWAS.

The accuracy of breeding value predictions was used to assess the performance of each prediction method for the validation set and was estimated as: $r_{GEBV,BV} = r_{GEBV,y} /h$; where $r_{GEBV,y}$ is the correlation of the breeding value predictions (EBV or GEBV) of animals in the validation data based on a given model and training data with the observed phenotypes of those animals, and $h$ is the square root of the narrow-sense heritability estimated with the ssGBLUP method [50–52]. The bias of the breeding value predictions from each model was estimated as the regression coefficient of the resistance phenotype (DAYS or STATUS) on the breeding value predictions (EBV or GEBV) for each validation set. Before calculating the regression coefficients, the predicted EBV and GEBV for the binary trait STATUS, which were estimated on the underlying scale of liability using the threshold model, were transformed to the observed scale using procedures that were described elsewhere [35]. Finally, the accuracy and bias reported are the averages across the five-fold CV sets, with 10 replications of the CV analysis to minimize the stochastic variation in the CV analysis.

## Results

### Heritability of IHNV resistance

Fish mortality rate in the IHNV challenge was equal to 0.36 in the YC 2016 families of this breeding population. Estimates of narrow-sense heritability were moderately high, with a range of values from 0.23 to 0.33 and from 0.25 to 0.28 for DAYS and STATUS, respectively (Table 1). Overall, estimates of heritability using genomic data (ssGBLUP; 0.23 and 0.25) were lower than those estimated with the pedigree-based model (PBLUP; 0.28 and 0.33). However, the ssGBLUP estimates had lower standard errors (SE) than the PBLUP estimates.

### QTL associated with IHNV resistance

Fifty-one 1-Mb windows that explained more than 1% EGV were detected (see Additional file 1: Table S1). These windows were used to define 21 QTL regions, of which 10 were categorized as QTL associated with IHNV resistance (EGV ≥ 2%) (QTL2.2, 4.1, 4.2, 6.1, 16.1, 17.1, 21.1, 25.1, 26.1 and 28.1) and 11 as QTL with suggestive association (1% ≤ EGV < 2%) (QTL 1.1, 2.1, 3.1, 5.1, 5.2, 8.1, 8.2, 14.1, 15.1, 25.2 and 29.1).

**Fig. 1:**
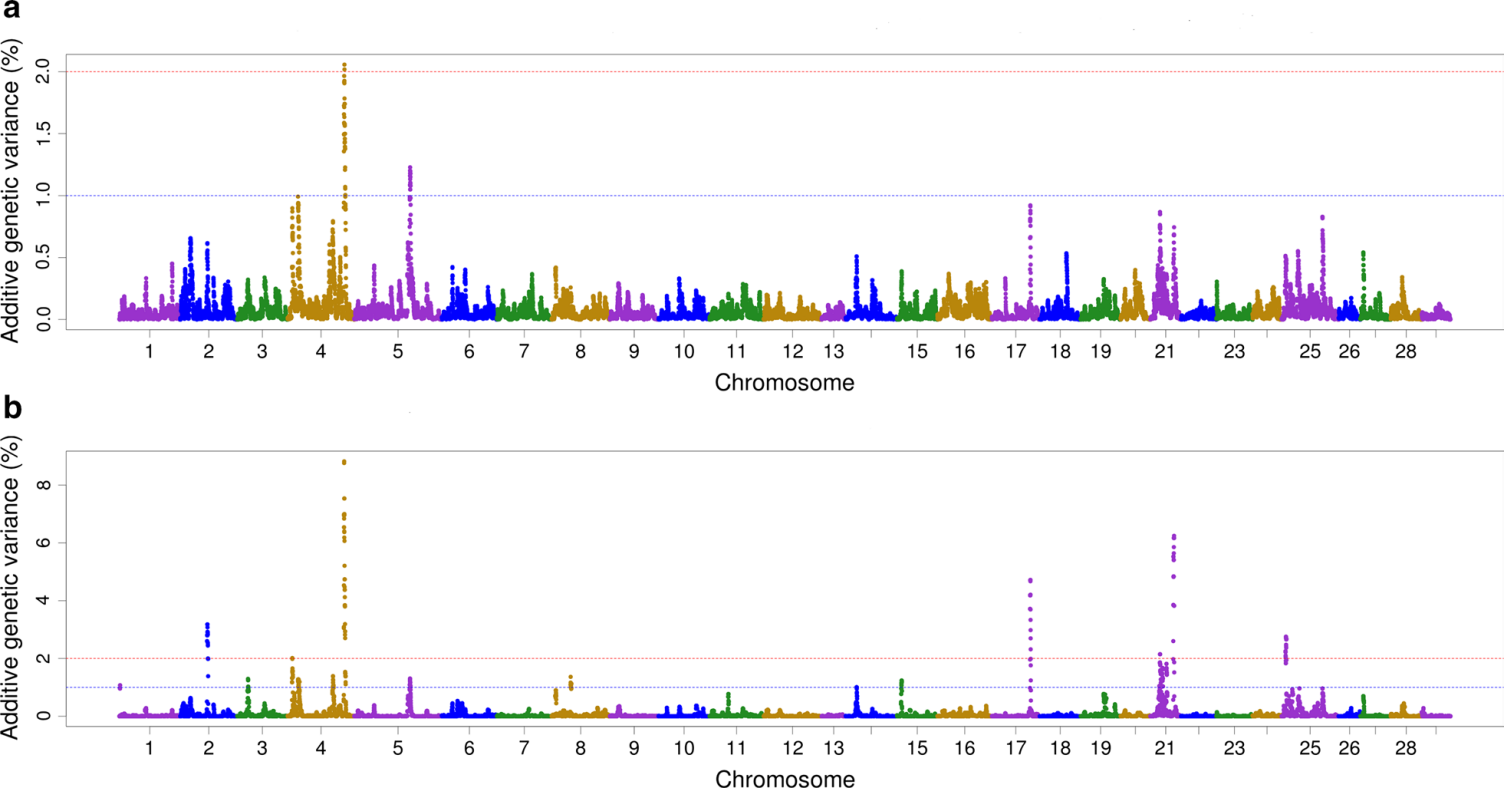
Manhattan plot showing the association of 1-Mb sliding-windows with IHNV survival DAYS. **a** GWAS using single-step GBLUP (ssGBLUP). **b** GWAS using weighted single-step GBLUP (wssGBLUP)

The IHNV resistance phenotypes of survival days and status are highly correlated. Hence, as expected, the QTL that were detected for STATUS were also detected for DAYS (see Additional file 1: Table S1). Manhattan plots from GWAS using wssGBLUP for DAYS (Fig. 1b) and STATUS (see Additional file 2: Figure S1b) show that the same QTL regions were detected for both traits (windows with EGV ≥ 2%). Thus, for the sake of clarity, in the remainder of this paper, we present results from GWAS for survival days and treat them as good proxies for QTL for the IHNV resistance trait.

**Fig. 2:**
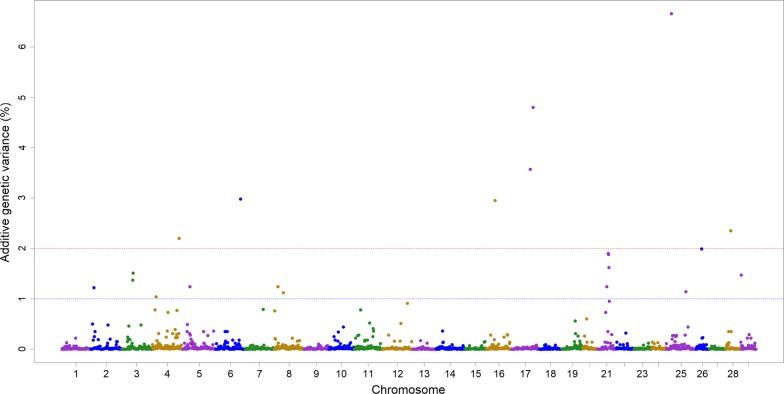
Manhattan plot showing the association of non-overlapping 1-Mb windows with IHNV survival DAYS based on single-step Bayesian multiple regression (ssBMR) with BayesB

Fifteen 1-Mb windows associated with IHNV resistance (EGV ≥ 2%) were detected using either the wssGBLUP or the ssBMR GWAS methods (Table 2). Of these windows, seven were detected with wssGBLUP and eight were detected with ssBMR. These 15 SNP windows cover 10 QTL regions associated with IHNV resistance that jointly explained up to 42% of the additive genetic variance when accounting only for the largest effect SNP window for each QTL region. The QTL 4.2 (EGV = 8.8%, Fig. 1b), 21.1 (EGV = 6.2%, Fig. 1b) and 25.1 (EGV = 6.7%, Fig. 2) had explained the highest proportions of genetic variance for IHNV resistance.

**Table 2:** Summary of QTL identified for IHNV survival DAYS

Omy	QTL^a^	GWAS method^b^	EGV (%)	Physical map (bp)		Window flanking SNP		SNPs per window
				Start	End	Start	End	
2	2.2	wssGBLUP	3.2	45,383,323	46,361,331	Affx-88954877	Affx-88928987	24
4	4.1	wssGBLUP	2.0	9,789,985	10,738,830	Affx-88917261	Affx-88929814	31
4	4.2	wssGBLUP	8.8	68,038,853	68,982,815	Affx-88923800	Affx-88922397	42
4	4.2	ssBMR	2.2	74,007,109	74,987,590	Affx-88939425	Affx-88928526	21
6	6.1	ssBMR	3.0	68,134,086	68,990,700	Affx-88929527	Affx-88939372	19
16	16.1	ssBMR	3.0	24,057,372	24,944,118	Affx-88954356	Affx-88951804	18
17	17.1	ssBMR	3.6	51,034,316	51,997,613	Affx-88955435	Affx-88944415	26
17	17.1	ssBMR	4.8	59,030,557	59,975,343	Affx-88934715	Affx-88947595	36
17	17.1	wssGBLUP	4.7	59,332,327	60,281,825	Affx-88919103	Affx-88944127	34
21	21.1	wssGBLUP	2.1	22,119,421	23,117,999	Affx-88932453	Affx-88916492	40
21	21.1	wssGBLUP	6.2	39,913,253	40,900,383	Affx-88932908	Affx-88907074	23
25	25.1	ssBMR	6.7	14,168,054	14,988,042	Affx-88923380	Affx-88924756	29
25	25.1	wssGBLUP	2.8	14,387,782	15,344,868	Affx-88930881	Affx-88908721	28
26	26.1	ssBMR	2.0	15,009,533	15,969,232	Affx-88931236	Affx-88925053	24
28	28.1	ssBMR	2.4	13,000,180	13,927,035	Affx-88912736	Affx-88929314	24

Offspring from year-class 2016 families from the nucleus breeding population of Clear Springs Foods, Inc.

^a^Summary of QTL regions including only those 1-Mb genomic windows with an explained additive genetic variance (EGV) higher than 2%

^b^GWAS was analyzed using weighted single-step GBLUP (wssGBLUP) and single-step Bayesian multiple regression (ssBMR); the ssBMR was performed using BayesB with the mixture parameter $\pi$ = 0.999

**Fig. 3:**
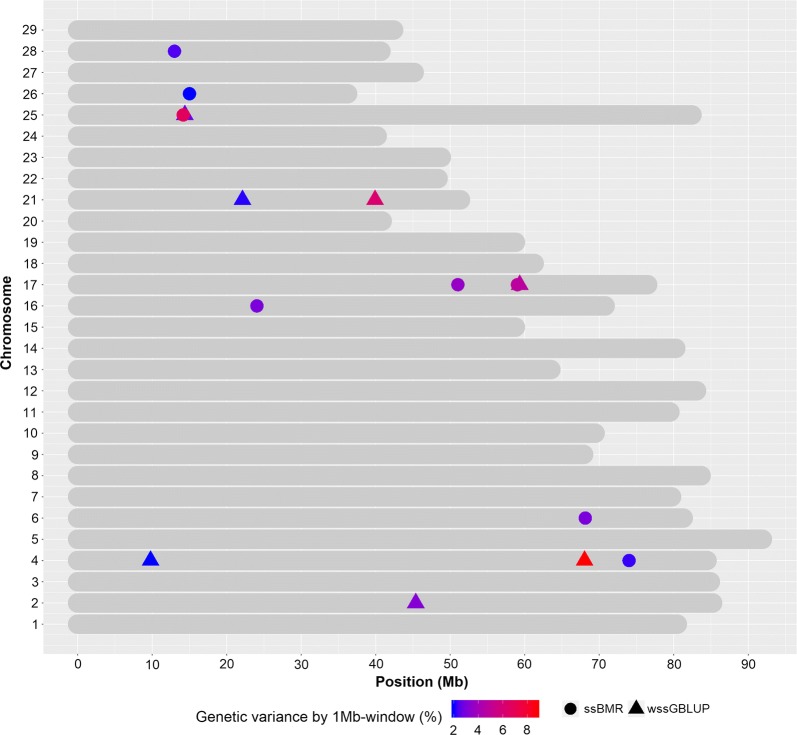
Co-localized 1-Mb QTL windows associated with IHNV survival DAYS (EGV ≥ 2%) detected with weighted single-step GBLUP (wssGBLUP) and single-step Bayesian multiple regression (ssBMR) with BayesB

Three QTL associated with IHNV resistance (4.2, 17.1, and 25.1) were detected by both GWAS methods (Table 2 and Fig. 3). Interestingly, the QTL 17.1 and 25.1 had the lowest PFP, i.e. 0.14 and 0.09, respectively, when detected with ssBMR; thus, they were the most significant QTL detected with the ssBMR method. Three QTL (2.2, 4.1 and 21.1) were detected only with wssGBLUP (Fig. 3), and four QTL (6.1, 16.1, 26.1 and 28.1) were detected only with ssBMR.

The physical map coordinates of the identified QTL regions for IHNV resistance (Table 2) were queried against the rainbow trout reference genome sequence annotation (RefSeq Accession GCF_002163495.1) [33]. In total, 774 predicted coding genes were identified within the QTL regions (see Additional file 3: Table S2).

The 1-Mb windows that were detected with EGV ≥ 1% for IHNV resistance using wssGBLUP and ssBMR are compared in Table S3 (see Additional file 4: Table S3). More windows were detected with ssBMR (21) than with wssGBLUP (17), which also translated to more QTL regions detected with ssBMR (16) than with wssGBLUP (12). Of those QTL regions, seven were detected by both GWAS methods, five were detected only by wssGBLUP and nine were detected only by ssBMR.

### Accuracy and bias of breeding value predictions for IHNV resistance

**Fig. 4:**
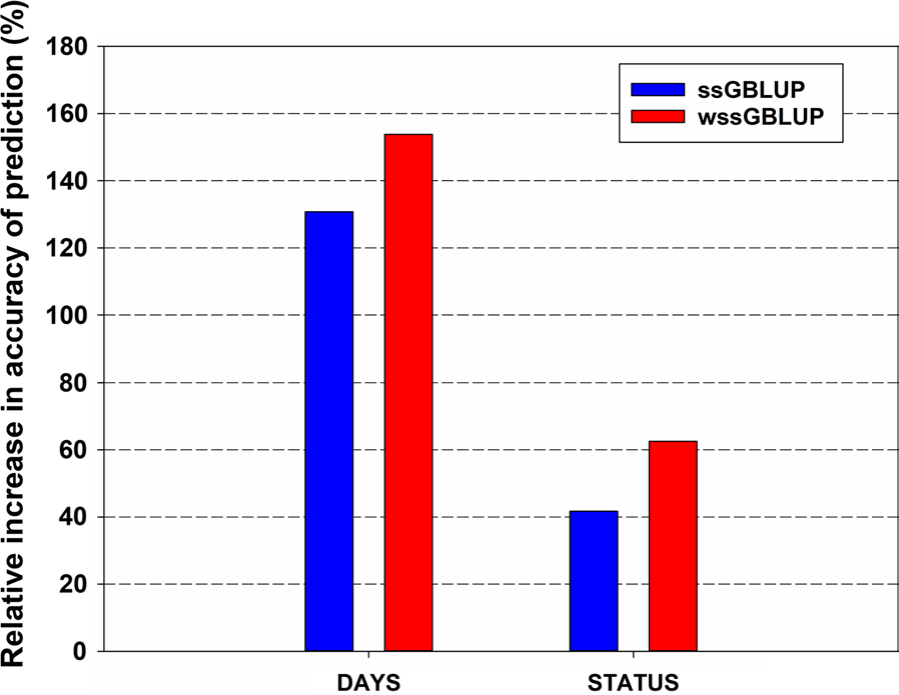
Relative increase in accuracy of genomic prediction for IHNV resistance (DAYS and STATUS) over pedigree-based BLUP (PBLUP). Genomic predictions were performed with single-step GBLUP (ssGBLUP) and weighted single-step GBLUP (wssGBLUP)

Overall, across the IHNV resistance phenotypes, the accuracy of breeding value predictions was higher for the genomic prediction models ssGBLUP (0.30–0.34) and wssGBLUP (0.33–0.39) than for the pedigree-based PBLUP model (0.13–0.24) (Table 3). Across prediction methods, the accuracies of predicted breeding values for survival STATUS (0.24–0.39) were higher than those for survival DAYS (0.13–0.33). The relative increase in accuracy of genomic predictions methods over the pedigree-based PBLUP is shown in Fig. 4. The accuracy of predictions with the wssGBLUP and ssGBLUP models substantially outperformed the pedigree-based PBLUP model, by 63 to 154% and 42 to 131%, respectively.

**Table 3:** Accuracy and bias of breeding value predictions for IHNV resistance using three methods

Method^a^	DAYS^b^		STATUS^c^	
	Accuracy^d^	Bias^e^	Accuracy^d^	Bias^e^
PBLUP	0.13	0.27	0.24	0.11
ssGBLUP	0.30	0.50	0.34	0.14
wssGBLUP	0.33	0.37	0.39	0.11

Offspring from year-class 2016 families from the nucleus breeding population of Clear Springs Foods, Inc.

^a^Animal breeding value predictions were performed using pedigree-based BLUP (PBLUP), single-step GBLUP (ssGBLUP) and weighted ssGBLUP (wssGBLUP)

^b^Discrete data survival days after disease challenge (DAYS). The accuracy and bias of animal merit predictions for DAYS were estimated using five-fold cross-validation analysis with 10 replications

^c^Binary data survival status (STATUS). The accuracy and bias of animal merit predictions for STATUS were estimated using five-fold cross-validation analysis with 10 replications

^d^Accuracy of breeding value predictions was estimated as the correlation of phenotypic records $y$ (DAYS or STATUS) with the animal merit predictions (EBV or GEBV) divided by the square root of heritability $\left( {h_{days}^{2} = 0.23; h_{status}^{2} = 0.25} \right)$

^e^Bias of breeding value predictions was estimated as the regression coefficient of phenotypic records $y$ (DAYS or STATUS) on the animal merit predictions (EBV or GEBV)

The bias of breeding value predictions was lower (i.e. closer to 1.0) for DAYS (0.27–0.50) than for STATUS (0.11–0.14) (Table 3). For survival DAYS, the genomic prediction methods ssGBLUP and wssGBLUP (0.37–0.50) had lower bias than pedigree-based PBLUP (0.27). For DAYS, ssGBLUP (0.50) had lower predictions bias than wssGBLUP (0.37). The predictions for STATUS had the highest bias and may not be valid because of the use of binary phenotype records when computing the regression coefficient in the CV analysis.

## Discussion

In this genome-wide association study, we detected 10 QTL regions that were associated with IHNV resistance and that together explained up to 42% of the additive genetic variance for IHNV resistance in a commercial rainbow trout breeding population. Only two of these QTL (17.1 and 25.1) were reported in previous studies (see Additional file 5: Table S4) [3, 10, 12] and the other eight QTL are novel (Table 2). We also determined that resistance against IHNV in rainbow trout is controlled by the oligogenic inheritance of a few loci with moderate effects (EGV = 2.0–8.8%) and a large-unknown number of loci each with small effects.

In addition, for the first time for this important disease resistance trait, we show that, based on empirical data, genomic prediction is expected to substantially outperform the classical pedigree-based predictions (PBLUP) in terms of accuracy. These results highlight the potential utility of genome-enabled selection for genetic improvement of resistance against IHNV in this commercial rainbow trout breeding population.

### Heritability of IHNV resistance

Estimates of heritability for IHNV resistance in our study were moderately (0.23–0.33) and somewhat lower than those reported previously using pedigree-based model in a different rainbow trout population [2]. Still, our estimates underline the potential for genetic improvement for IHNV resistance in this commercial rainbow trout population through selective breeding. Our heritability estimates based on genomic data (ssGBLUP) were consistently lower than those based on PBLUP. However, estimates from ssGBLUP had much lower standard errors than those estimated with PBLUP. Interestingly, our estimate of heritability for survival STATUS using genomic data (0.25) was also lower than the heritability estimate for IHNV mortality using a pedigree-based model by Brieuc et al. [2] (0.38). With the pedigree-based PBLUP model, there is a confounding between the phenotype and the Mendelian sampling term, and we expect this confounding to be less of a problem when using genomic data with ssGBLUP, since the covariances between Mendelian sampling terms of relatives can be quantified based on the proportion of the genome that they share [53, 54].

### QTL associated with IHNV resistance

In this study, we did not detect large-effect QTL for IHNV resistance, which suggests that marker-assisted selection is not likely to be an effective strategy for improving the genetic resistance of rainbow trout against IHNV in this specific commercial population. However, further fine-mapping of the detected IHNV-QTL regions and subsequent identification of putative candidate genes or causative mutations would be valuable for advancing the understanding of the mechanisms of genetic resistance to IHNV in rainbow trout and the underlying biology of host–pathogen interactions. This can be achieved by genotyping a large number of SNPs at positions within and near the major QTL regions, and by re-sequencing highly characterized IHNV resistant and susceptible fish, as was effectively done in the search for the IPNV resistance gene in Atlantic salmon [55]. We generated an initial preliminary list of positional candidate genes for the IHNV-QTL regions by cross-examining the rainbow trout reference genome sequence annotation (RefSeq Accession GCF_002163495.1) [33]. Overall, we identified 774 protein-encoding features located within the QTL identified for IHNV resistance (see Additional file 3: Table S2), which are now available for future analyses and research efforts that are beyond the scope of this study.

Previous QTL mapping studies have reported 36 QTL for IHNV resistance in rainbow and steelhead trout backcross populations (see Additional file 5: Table S4) [3, 9–12] but most of those QTL were not detected in our study. Only two out of the 10 QTL identified in our study were previously reported, i.e. QTL17.1 [3, 10] and 25.1 [12], which interestingly had the lowest PFP in our study. Thus, we report eight novel unpublished QTL for IHNV resistance in rainbow trout. This poor overlap of the QTL detected in our study with those from previous reports is caused, in part, by differences in the genetic background and in population or sampling structures between the studies.

It is worth noting that Campbell et al. [9] detected 19 RAD SNPs associated with IHNV resistance in this same CSF commercial breeding population, although using data from two generations previous to the current data (see Additional file 5: Table S4). Surprisingly, none of their reported QTL overlap or were close to the 10 QTL regions detected here. These conflicting results in QTL mapping can be explained by several reasons including: (1) this population has not been closed and additional families were introduced to the breeding population in the past two generations; (2) QTL effects can be population- and/or family-specific, with unique extent/phase of linkage and extent of LD between QTL and marker alleles; and (3) the previously detected QTL may represent false positive results due to limitations and weaknesses on experimental design and methods used for data analyses.

### Comparison of GWAS methods

The use of correct statistical models and computer algorithms is crucial for uncovering the underlying genetic basis of resistance to diseases with multifactorial inheritance using GWAS. In this study, we scanned the genome for loci that were associated with IHNV resistance using two single-step multiple regression based GWAS methods that estimate the effect of all markers simultaneously, thus accounting for LD between neighboring loci [15, 16, 56]. A unique feature of these multiple regression single-step GWAS methods is that, in the analysis, they employ all available pedigreed animals with genotype and/or phenotype records. Thus, they are expected to have higher power of QTL detection than GWAS methods that do not use a single-step approach and test for association using one-marker at a time without accounting for LD between linked loci and without using phenotypes on non-genotyped relatives. Of the 10 QTL regions identified for IHNV resistance, only three were detected by both GWAS methods, and seven were detected by only one method, which supports the utility of using different GWAS algorithms to uncover the QTL associated with a complex disease resistance trait.

We also evaluated GWAS models that assume a purely polygenic inheritance for IHNV resistance and a normal distribution of the marker effects and found that ssGBLUP (Fig. 1a) and RR-BLUP [20, 21, 57] did not result in enough power to detect QTL for IHNV resistance (results not shown). These results confirmed the superiority of GWAS methods such as ssBMR and wssGBLUP, which assume that the genetic variance of a trait is explained by a reduced number of QTL with moderate-to-large effects, instead of purely polygenic inheritance [29, 58, 59]. Furthermore, these results support the importance of testing several GWAS methods when attempting, for the first time, to elucidate the underlying genetic basis of resistance to complex diseases such as IHNV in rainbow trout.

The marginal difference in power of QTL detection between the two single-step based GWAS methods is due to differences in their assumptions. The Bayesian variable selection model BayesB that was performed with ssBMR was shown to be robust to population structure and to cryptic family relationships in GWAS and GS with admixed populations [18], and also more powerful than standard mixed linear GBLUP-based models when the trait under study is controlled by few genes/loci with a moderate to large effect and many loci with a small effect, i.e. for an oligogenic inheritance trait [35, 59, 60].

We also found that ssBMR detected a larger number of QTL (7) associated with DAYS (Fig. 2) than the standard BayesB method (4) [15, 45] (see Additional file 6: Figure S2), which uses only individuals that have both phenotype and genotype records ($n$ = 992), because ssBMR used all the animals ($n$ = 4987) available in this study [28, 29], including animals that had only phenotype records ($n$ = 3995).

### Accuracy and bias of breeding value predictions

Across the evaluated methods, the accuracy of breeding value predictions for STATUS were higher than those for DAYS (Table 3). However, the predictions for DAYS were less biased than those for STATUS. The computed bias of predictions for STATUS when using the CV analysis is incorrect and, consequently, was the most biased due to extreme-phenotype problems in which all binary observations within class variables are identical, i.e. each animal has one binary survival phenotype record of either 0 or 1 [52]. The incorrect bias estimation for breeding value predictions when using binary data in CV analysis is known and it is an area of active research [61–63], which is outside the scope of this study. However, this problem can be circumvented by assessing the accuracy and bias of breeding value predictions using progeny performance data (i.e. offspring survival rate per evaluated family), as we have shown elsewhere [35, 41]. We are in the process of generating those progeny performance records for a future report.

The accuracy of pedigree-based EBV predictions for STATUS was 0.24 when performing CV analysis using IHNV records from the 2016 CSF families (Table 3). Remarkably, this accuracy was quite close to the average historical accuracy of EBV predictions for IHNV resistance (0.25) (see Additional file 7: Table S5). This historical accuracy of EBV predictions was estimated by using progeny performance data from IHNV evaluations performed over five generations in the CSF breeding population, as the correlation of mid-parent EBV with the offspring survival rate from each evaluated family in each generation.

Overall, accuracies of genomic predictions for DAYS and STATUS with wssGBLUP (0.33–0.39) and ssGBLUP (0.30–0.34) were higher than those from the pedigree-based PBLUP model used with both CV analysis (0.13–0.24) and historical progeny performance data (0.25). Therefore, these results show the high potential for effective genetic improvement of IHNV genetic resistance using genome-enabled selective breeding in this commercial rainbow trout population.

## Conclusions

Our comprehensive genome-wide scan for QTL for IHNV resistance revealed that genetic resistance to IHNV in this commercial rainbow trout breeding population is controlled by the oligogenic inheritance of up to 10 QTL with moderate effects and many loci with small effects. Taken together, our results suggest that whole-genome-enabled breeding value prediction models will be more effective than the conventional pedigree-based prediction method or the marker-assisted selection approach for improving genetic resistance of rainbow trout against IHNV in this economically important breeding population.

## Supplementary information

**Additional file 1: Table S1.** Summary of the genomic windows with an explained genetic variance (EGV) higher than 1% for IHNV resistance phenotypes (DAYS and STATUS) detected in the Clear Springs Foods, Inc. rainbow trout breeding population. The data provided represents all the genomic windows that had an EGV higher than 1% when performing GWAS for IHNV survival DAYS and STATUS using weighted single-step GBLUP (wssGBLUP) and single-step Bayesian multiple regression (ssBMR) in the Clear Springs Foods, Inc. rainbow trout breeding population.

**Additional file 2: Figure S1.** Manhattan plot showing the association between 1-Mb sliding-windows and IHNV survival STATUS in Clear Springs Foods, Inc. rainbow trout breeding population: (a) GWAS using ssGBLUP. (b) GWAS using wssGBLUP. These plots represent the explained additive genetic variance for IHNV survival STATUS by each 1-Mb-sliding window that was tested along the rainbow trout genome with single-step GBLUP (ssGBLUP) and weighted single-step GBLUP (wssGBLUP) in the Clear Springs Foods, Inc. rainbow trout breeding population.

**Additional file 3: Table S2.** List of the 774 genes that are located within the 1-Mb windows that are associated with IHNV resistance in the Clear Springs Foods, Inc. rainbow trout breeding population. This data represents a list of 774 genes that are located in the QTL windows associated with IHNV resistance generated by query of the rainbow trout reference genome sequence annotation (RefSeq Accession GCF_002163495.1).

**Additional file 4: Table S3.** Comparison of genomic windows with an explained genetic variance (EGV) higher than 1% for IHNV survival DAYS and QTL regions detected with wssGBLUP and ssBMR in the Clear Springs Foods, Inc. rainbow trout breeding population. The data provided represent all the genomic windows that had an EGV higher than 1% when performing GWAS for IHNV survival DAYS using weighted single-step GBLUP (wssGBLUP) and single-step Bayesian multiple regression (ssBMR) in the Clear Springs Foods, Inc. rainbow trout breeding population. Table S3 was built to perform a fair comparison between wssGBLUP and ssBMR GWAS methods.

**Additional file 5: Table S4.** Summary of QTL for IHNV resistance reported in previous studies in rainbow trout populations. The data provided represent a summary of all reported QTL for IHNV resistance in past QTL/GWAS studies performed in rainbow trout populations. This table provides information on experimental variables including sample size, number of markers, disease phenotype, method of analysis, software, location of reported QTL on genetic and physical maps, and statistics such as nominal *P* value and LOD score for the reported QTL.

**Additional file 6: Figure S2.** Manhattan plot showing the association between 1-Mb exclusive-windows and IHNV survival DAYS using the BayesB method in Cold Springs Food, Inc. rainbow trout breeding population. These plots represent the explained additive genetic variance for IHNV survival STATUS by each 1-Mb-exclusive window when using the standard BayesB method with complete data (i.e., 992 fish that had both phenotype and genotype records) in the Clear Springs Foods, Inc. rainbow trout breeding population.

**Additional file 7: Table S5.** Historical accuracy of pedigree-based EBV predictions for IHNV resistance in Clear Springs Foods, Inc. rainbow trout breeding population. These data represent the historical accuracy of animal merit predictions that were estimated with the pedigree-based PBLUP model using empirical progeny survival records from IHNV challenges performed in year-class 2008, 2010, 2012, 2014 and 2016 families in the Clear Springs Foods, Inc. rainbow trout breeding population. The accuracy of predicted EBV are estimated as the correlation coefficient of mid-parent, sire or dam EBV with their offspring or progeny survival rate.

## Data Availability

The datasets supporting the conclusions of this research article are included within the article and its additional files.
