# Supplementary figures and images for: Genome-wide association analysis and accuracy of genome-enabled breeding value predictions for resistance to infectious hematopoietic necrosis virus in a commercial rainbow trout breeding population
Source: Genet Sel Evol. 2019 Aug 28;51:47. doi: 10.1186/s12711-019-0489-z (PMC6712688; doi:10.1186/s12711-019-0489-z)

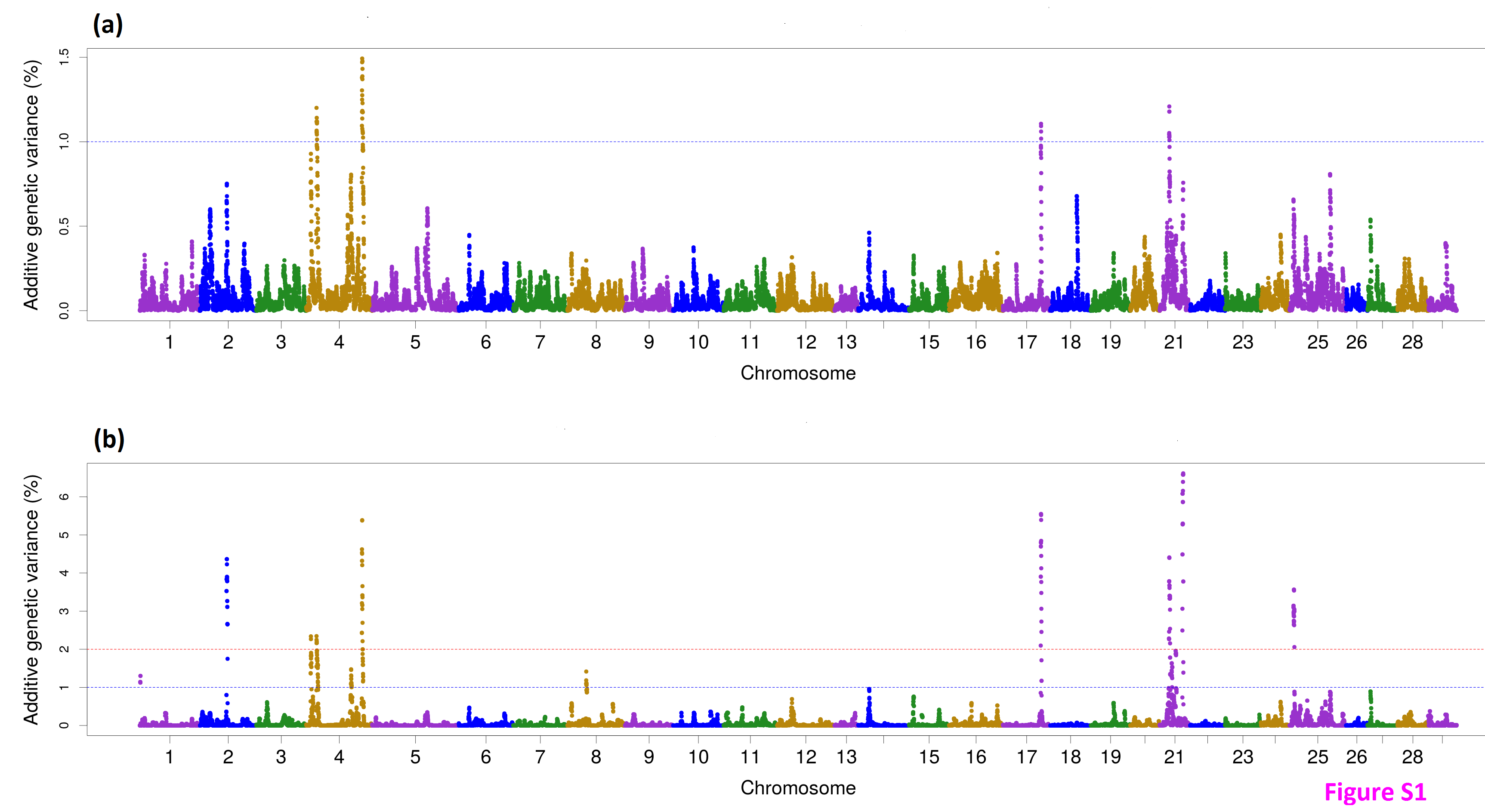

Supplement: Supplementary file 2 — Additional file 2: Figure S1. Manhattan plot showing the association between 1-Mb sliding-windows and IHNV survival STATUS in Clear Springs Foods, Inc. rainbow trout breeding population: (a) GWAS using ssGBLUP. (b) GWAS using wssGBLUP. These plots represent the explained additive genetic variance for IHNV survival STATUS by each 1-Mb-sliding window that was tested along the rainbow trout genome with single-step GBLUP (ssGBLUP) and weighted single-step GBLUP (wssGBLUP) in the Clear Springs Foods, Inc. rainbow trout breeding population. [file 12711_2019_489_MOESM2_ESM.tiff]

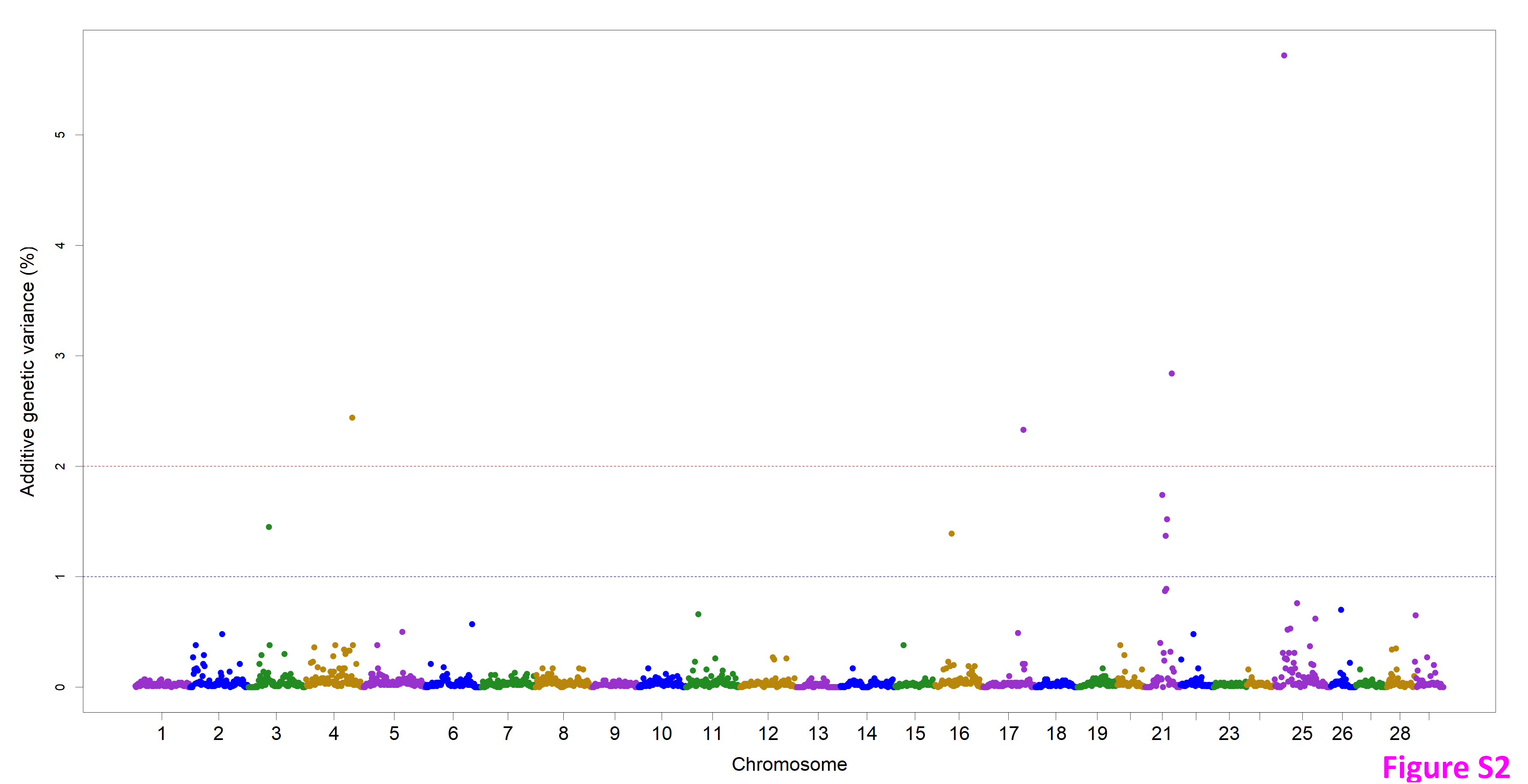

Supplement: Supplementary file 6 — Additional file 6: Figure S2. Manhattan plot showing the association between 1-Mb exclusive-windows and IHNV survival DAYS using the BayesB method in Cold Springs Food, Inc. rainbow trout breeding population. These plots represent the explained additive genetic variance for IHNV survival STATUS by each 1-Mb-exclusive window when using the standard BayesB method with complete data (i.e., 992 fish that had both phenotype and genotype records) in the Clear Springs Foods, Inc. rainbow trout breeding population. [file 12711_2019_489_MOESM6_ESM.tiff]
